# The In Vitro and In Vivo Anticancer Effect of Photomed for Photodynamic Therapy: Comparison with Photofrin and Radachlorin

**DOI:** 10.3390/cimb45030162

**Published:** 2023-03-17

**Authors:** Jieun Kim, Johyun Kim, Heewon Yoon, Yoon-Jee Chae, Kiyon Rhew, Ji-Eun Chang

**Affiliations:** 1 College of Pharmacy, Seoul National University, Seoul 08826, Republic of Korea; 2 College of Pharmacy, Dongduk Women’s University, Seoul 02748, Republic of Korea; 3 College of Pharmacy, Woosuk University, Wanju-gun 55338, Republic of Korea

**Keywords:** photodynamic therapy, cancer treatment, novel photosensitizer, chlorin derivative, pyrazole

## Abstract

To overcome the limitation of conventional cancer treatments, photodynamic therapy (PDT) has been introduced as another treatment option. PDT provides a non-invasive, non-surgical way with reduced toxicity. To improve the antitumor efficacy of PDT, we synthesized a novel photosensitizer, a 3-substituted methyl pyropheophorbide-a derivative (Photomed). The purpose of the study was to evaluate the antitumor effect of PDT with Photomed comparing with the clinically approved photosensitizers Photofrin and Radachlorin. The cytotoxicity assay against SCC VII cells (murine squamous cell carcinoma) was performed to determine whether Photomed is safe without PDT and whether Photomed is effective against cancer cells with PDT. An in vivo anticancer efficacy study was also performed using SCC VII tumor-bearing mice. The mice were divided into small-tumor and large-tumor groups to identify whether Photomed-induced PDT is effective for not only small tumors but also large tumors. From in vitro and in vivo studies, Photomed was confirmed to be (1) a safe photosensitizer without laser irradiation, (2) the most effective photosensitizer with PDT against cancers compared to Photofrin and Radachlorin and (3) effective with PDT in treating not only small tumors but also large tumors. In conclusion, Photomed may contribute as a novel, potential photosensitizer for use in PDT cancer treatment.

## 1. Introduction

According to statistics about the global burden of disease, cancer was ranked as the second highest burden of disease globally [1]. In spite of advancements in treatment modalities, cancer treatment remains a major challenge. Currently, surgery, radiation therapy and chemotherapy are the most commonly selected therapies for cancer treatments. Other treatments such as immunotherapy, hormone therapy, targeted therapy or stem cell therapy are infrequently used compared with major ones. Surgery is the very commonly used cancer treatment option. It is chosen for the first treatment option for early stages of various solid tumors such as lung, liver, breast and stomach cancers. It simply involves removing the tumor tissues; however, sometimes adjuvant radiation therapy or adjuvant chemotherapy is needed for a complete cure [2]. Radiation therapy is often used in combination with other treatments, especially surgery. Currently, it is often combined with immunotherapy since it is known to promote immune system in the body [3]. Radiotherapy is commonly used for certain cancer types including leukemia, lymphomas and also solid tumors. However, it is limited by its inadequacy in the treatment of metastatic cancers as it is not possible to give radiation to different parts of the body at the same time. In addition, radiotherapy provides various side effects during and after treatment such as fatigue, diarrhea, skin toxicity, weight loss and hair loss [4]. Chemotherapy is selected for the most standard treatment option for many cancers especially, metastatic cancers [5]. Chemotherapy may be the last treatment option for cancer patients who are not suitable candidates for any other cancer treatments. Based on various patients’ factors, chemotherapy may be prescribed solely or in combination with other treatment options. The choice of treatment is dependent on various factors including the patient’s condition, quality of life, life expectancy, cancer type, cancer stage and cancer location [6]. Although chemotherapy is a strong anticancer treatment option, it is also well-known to cause severe side effects which include hair loss, pain, mouth sores, nausea, vomiting, diarrhea and rashes. Since the cytotoxic chemotherapy agents attack both cancer cells and rapid proliferating normal cells, a large number of unexpected side effects often come out [7].

To overcome these limitations of conventional cancer treatments, various treatments have been established. Photodynamic therapy (PDT) has emerged as a promising alternative cancer treatment option. A photosensitizer, light and oxygen are the three main components for PDT. When the special agent called a photosensitizer is injected into the cancer patient, it becomes distributed in the patient’s body, and it selectively accumulates in the tumor site. The photosensitizer produces reactive oxygen species (ROS) such as singlet oxygen (^1^O_2_), superoxide anions (∙O_2_^−^) and hydroxyl radicals (∙OH) with the proper wavelength of light. Singlet oxygen especially induces apoptosis and necrosis of the tumor [8]. The ways how PDT plays a role in cancer treatment are (1) that ROS directly attack cancer cells, (2) that PDT leads to tumor-associated vascular damage and (3) that PDT facilitates immune response [9]. When compared with conventional cancer treatments, PDT shows several advantages including: (1) it is a less invasive method when compared to surgery or radiation therapy; (2) it only takes a few minutes to be irradiated; (3) it provides tumor selectivity which may lead to reduced side effects that result from attacking normal cells; (4) the treatment may be repeated for several times at the same location if necessary; (5) there is little or no scar after treatment; (6) it easily combines with any other treatments such as chemotherapy, targeted therapy, immunotherapy or radiation therapy. Taken all together, PDT may provide higher therapeutic efficacy against tumors compared with the conventional therapies.

To improve the antitumor efficacy of PDT, various research is ongoing. Conjugate targeting moieties to photosensitizers, adopting nanoformulation to photosensitizers and combining other cancer therapies to PDT, are representative novel approaches for PDT. We focused on developing novel photosensitizers. Some properties are suggested to make the ideal photosensitizers including tumor localization, minimal dark toxicity, minimal photosensitivity, high ROS production and maximum light toxicity [10]. The first-generation photosensitizers, which are hematoporphyrin and its derivatives, were introduced in the 1970s. Photofrin was the first photosensitizer to be approved in the use of PDT for the cancer treatment. In the late 1980s, the second-generation photosensitizers were developed to overcome the limitations of the first-generation photosensitizers, such as skin photo-sensitivity and low tissue penetration. The second-generation photosensitizers include chlorins, benzoporphyrins, phthalocyanines and naphthalocyanines as well as their derivatives. These compounds provide deeper tissue penetration, higher production of singlet oxygen, higher tumor selectivity, quicker tumor uptake (shortening the time interval between photosensitizer administration and light irradiation) and more rapid clearance from the normal tissues, reducing photosensitization of normal tissues compared to the first generation photosensitizers [11,12]. Recently, novel chlorin photosensitizers such as monocationic chlorin photosensitizer [13], chlorin conjugates containing D-galactose or L-arginine [14] and chlorophyll-a derivatives containing oligoethylene glycol substituents [15] have been introduced with their improved antitumor efficacy compared to established cholorins.

In this study, we synthesized new chlorin derivatives since chlorin derivatives showed several beneficial PDT effects compared to porphyrin derivatives. We adopted a pyrazole system on the peripheral position of chlorin derivative. The synthesis of pyra-zolechlorins was reported to provide further functions for tetrapyrrole structures having acetylacetonate substituents [16]. The peripheral heterocyclic systems proceeding from acetylacetone derivatives of chlorin derivatives may enhance the biological and pharmaceutical activities of chlorin derivatives themselves. In addition, the pyrazoles play a role as potent anticancer agents against various cancers [17,18]. In the previous study, various methyl pyropheophorbide-a (MPPa) derivatives that possess heterocyclic aromatic substituents or pyrazole moieties on peripheral position-3 were synthesized [19]. Among the 3-substituted MPPa derivatives, 3-[(3,5-Dimethyl-1H-pyrazole-4-yl)methyl]-3-devinyl pyropheophorbide-a methyl ester (Photomed) showed effective accumulation in tumor sites after intravenous injection; moreover, it demonstrated the strong PDT induced antitumor efficacy after intratumoral injection. The goal of the present study was to evaluate the antitumor effect of PDT after intravenous injection of Photomed which will be chosen for the clinical administration route. More importantly, the PDT efficacy of Photomed was compared with Photofrin (porfimer sodium) and Radachlorin (sodium salts of chlorin e6) which are clinically approved photosensitizers for PDT against various cancers. In addition, the study was performed with not only small-tumor groups (tumor size ranging from 100 to 200 mm^3^) but also large-tumor groups (tumor size ranging from 200 to 300 mm^3^) to investigate the efficacy of PDT according to the tumor size. 

## 2. Materials and Methods

### 2.1. Materials

Photofrin and Radachlorin were kindly provided by Dr. I&B (Daejeon, Republic of Korea). Phosphate buffered saline (PBS), Dulbecco’s modified eagle medium (DMEM), fetal bovine serum (FBS) and trypsin-EDTA (TE) were purchased from Welgene (Daegu, Republic of Korea). A 100X antibiotic–antimycotic solution was obtained from Gibco (Carlsbad, CA, USA). All the other reagents regarding synthesis were purchased from Sigma-Aldrich (St. Louis, MO, USA) and TCI (Tokyo, Japan).

### 2.2. Synthesis of Photomed

Photomed was synthesized according to the previously described method [19,20]. MPPa was selected as a starting compound, and then MPPd, PO100, PA100 and Photomed were synthesized in order.

### 2.3. Singlet Oxygen Photogeneration Analysis

A total of 50 μM of 1,3-diphenylisobenzofuran (DPBF) solution with PBS, 1 μM of methylene blue or photosensitizers (Photofrin, Radachlorin and Photomed) was loaded into a 96-well plate (SPL Life Sciences, Gyeonggi-do, Korea) and irradiated (5 mW/cm^2^, 400 s) with the proper wavelength (630 nm for Photofrin and 662 nm for Radachlorin and Photomed) of PDT lasers (Dr. I&B). Remaining DPBF was measured at 418 nm absorbance by the microplate reader (Spark^®^, Tecan, Männedorf, Switzerland). Every experiment was tested in triplicate.

### 2.4. In Vitro Cytotoxicity Test 

SCC VII cells (murine squamous cell carcinoma), which were kindly supplied by Gwangju Institute of Science and Technology, were seeded in 24-well cell culture plates (SPL Life Sciences, Gyeonggi-do, Korea) at a density of 1.0 × 10^5^ cells/well in DMEM medium containing 10% (*v*/*v*) FBS and 1% (*v*/*v*) 100× antibiotic–antimycotic solution. The seeded cells were incubated for 24 h at 37 °C in a humidified 5% CO_2_ and 95% air environment for cell attachment. The medium was removed, and the fresh medium containing various concentrations (0, 0.1, 0.2 0.25, 0.4 and 0.5 μM) of photosensitizers (Photofrin, Radachlorin and Photomed) was treated. After 4 or 24 h of incubation under dark condition, the drugs were discarded, and the cells were washed twice with cold PBS to eliminate remaining photosensitizers. Then, the fresh medium was added into each well. For the “PDT group”, the cells were irradiated (5 mW/cm^2^, 400 s) with the proper wavelength (630 nm for Photofrin and 662 nm for Radachlorin and Photomed) of PDT lasers. Every irradiation was performed in the clean bench in order to prevent contamination of the cells. For the “dark group”, no irradiation was performed. Both groups were incubated for 24 h in the dark. The cells were washed twice with cold PBS, and the cell viability was detected using a Cell Counting Kit-8 (CCK-8) (Dojindo Molecular Technologies, Rockville, MD, USA) [21]. Totals of 10 μL of CCK-8 solution and 100 μL of DMEM mixture were added to each well, and then the cells were incubated in the dark for another 2 h. Lastly, the samples were placed on the 96-well plate, and the absorbance of OD at 450 nm was measured by the microplate reader. Every experiment was tested in triplicate.

Microscopic analysis was also performed to visualize apoptotic cells caused by PDT. The cells were treated with 0.5 μM of photosensitizers (Photofrin, Radachlorin and Photomed) and incubated for 24 h under dark conditions. After washing with cold PBS twice, the cells were irradiated (5 mW/cm^2^, 400 s) with the proper wavelength (630 nm for Photofrin and 662 nm for Radachlorin and Photomed) of PDT lasers (PDT group), or no irradiation was given (dark group). After 24 h of incubation, the cells were double stained with Hoechst 33342 and Annexin V-FITC. Then, the stained cells were washed with cold PBS twice and observed under an inverted fluorescence microscope (Nikon-Eclipse TE2000-U, Nikon, Tokyo, Japan) fitted with a high-pressure mercury lamp (C-SHG1, Nikon). 

### 2.5. In Vivo Anticancer Efficacy Study

BALB/C male nude mice (7 weeks, 20–22 g) were obtained from Orientbio (Gyeonggi-do, Republic of Korea). Some 1 × 10^6^ SCC VII cells were subcutaneously injected into the left flanks of mice to establish tumor models. After tumor induction, the mice were divided into the small-tumor group (tumor size ranging from 100 to 200 mm^3^) and the large-tumor group (tumor size ranging from 200 to 300 mm^3^). Then, each group was randomly divided into 4 groups (*n* = 5 per group) and injected twice (on day 0 and 7) with PBS or various photosensitizers (Photofrin, Radachlorin and Photomed) via tail vein. The concentration of photosensitizers was all set to be 1 mg/kg, and 3 h after each injection, tumors were irradiated (400 mW/cm^2^, 500 s) twice (on day 0 and 7) with the proper wavelength (630 nm for Photofrin and 662 nm for Radachlorin and Photomed) of PDT lasers. Every irradiation was performed on the heating pad (37 °C) in order to maintain the body temperature of the mice during the treatment. After irradiation, the animals were housed in the dark for 24 h to avoid the influence of any other light source. The tumor volume, body weight and tumor surface change in every mouse were monitored for 3 weeks. The tumor size was measured with calipers, and the tumor volume (mm^3^) was calculated using the following formula: (length × width^2^)/2. To observe the necrosis or apoptosis from the tumor sites, hematoxylin and eosin (H&E) staining was performed on day 10. The formalin-fixed and paraffin-embedded (FFPE) representative tumor tissues from every group were prepared.

### 2.6. Statistical Analysis 

All data were expressed as the mean ± standard deviation (SD). The statistical significance between the groups was determined using the Student’s *t*-test for 2 groups. The differences were considered significant when *p* values were less than 0.05 (*p* < 0.05) in every analysis.

## 3. Results and Discussion

### 3.1. Synthesis of Photomed

**Figure 1:**
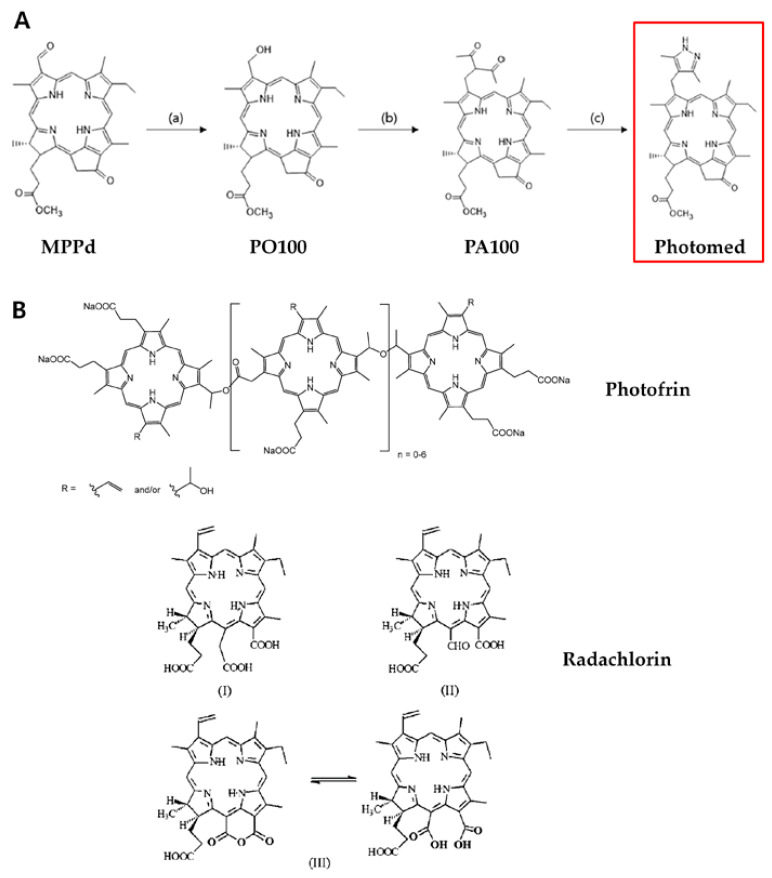
(**A**) Synthesis of Photomed. (a) *t*-BuNH_2_BH_3_, CH_2_Cl_2_, for 12 h, at room temperature, under nitrogen. (b) Acetyl acetone, Zn(OAc)_2_, for 1 h at 60 °C, for 1.5 h at 110 °C, 6 N HCl for 20 min. (c) Hydrazine hydrate, EtOH, CH_2_Cl_2_, for 2 h, at room temperature. (**B**) Structures of Photofrin [24] and Radachlorin: chlorin e6 (I), chlorin p6 (II) and purpurin (III) [23].

Photomed (3-[(3,5-Dimethyl-1H-pyrazole-4-yl)methyl]-3-devinyl pyropheophorbide-a methyl ester) was synthesized starting from the MPPa compound. The scheme of the Photomed synthesis process is shown in Figure 1A. The characterizations of Photomed were reported before [19]. From the previous study, new heteroaromatic-substituted chlorin derivatives (PO100, PA100 and Photmed) were evaluated for their photodynamic therapeutic efficacy against cancer cells and among them, Photomed showed potency as an effective photosensitizer [19]. For this reason, Photomed was selected to compare the photodynamic therapeutic effect with clinically approved photosensitizers (Photofrin and Radachlorin) in this study. Photofrin (porfimer sodium) is a hematoporphyrin derivative which belongs to the first generation of photosensitizers. It was the first US Food and Drug Administration (FDA) approved photosensitizer for treating cancers with PDT [22]. Radachlorin is a chlorin derivative which is a mixture of 90–95% sodium salts of chlorin e6, 5–7% chlorin p6 and 1–5% purpurin 5 [23]. It is one of the most widely used second-generation photosensitizers. The structures of Photofrin and Radachlorin are also introduced in Figure 1. When compared to the first-generation photosensitizers, the second-generation photosensitizers have several advantages. While the first-generation photosensitizers have their absorption band at 630 nm, the second generation photosensitizers show their absorption band at 650–850 nm with an increased molar extinction coefficient which allows for deeper tissue penetration. In addition, the second-generation photosensitizers have quicker pharmacokinetics, which means fast tumor uptake, high tumor selectivity and fast clearance. In this manner, the second-generation photosensitizers may shorten the treatment period and also reduce the unwanted adverse events such as skin photosensitivity. Moreover, higher production of singlet oxygen may lead to increased therapeutic efficacy for the second-generation photosensitizers. Photomed is a heteroaromatic-substituted chlorin derivative having pyrazole moieties at the 3-position. This strategy may increase biological and pharmaceutical activities of chlorin derivatives themselves and enhance the therapeutic efficacy against cancers.

### 3.2. Singlet Oxygen Photogeneration Analysis

To determine the production of singlet oxygen (^1^O_2_) after irradiation, a DPBF assay was performed. DPBF is a selective singlet oxygen acceptor, and when it binds with single oxygen, the generated diketone structure decreases the absorbance of DPBF [20]. Methylene blue was selected as a standard singlet oxygen sensitizer.

**Figure 2:**
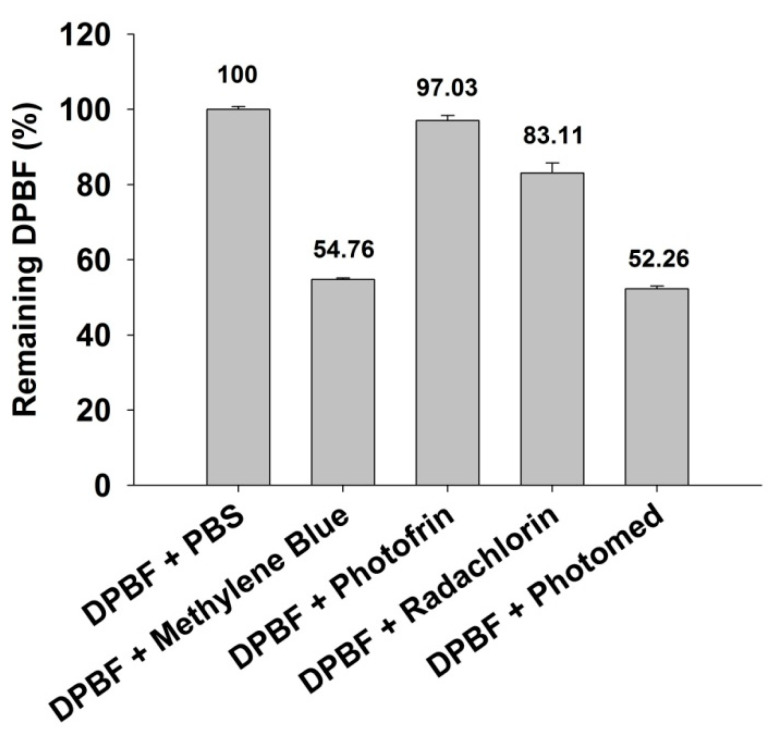
Singlet oxygen photogeneration assay. PBS, 1 μM of methylene blue or photosensitizers (Photofrin, Radachlorin and Photomed) were added to 50 μM of 1,3-diphenylisobenzofuran (DPBF) solution and irradiated (5 mW/cm^2^, 400 s) with the proper wavelength (630 nm for Photofrin and 662 nm for Radachlorin and Photomed) of PDT lasers. Remaining DPBF was measured at 418 nm absorbance. Data are presented as means ± SD (*n* = 3).

As shown in Figure 2, Photomed (52.26 ± 0.73% of remaining DPBF) displayed remarkable singlet oxygen generation which was comparable to methylene blue (54.76 ± 0.34% of remaining DPBF). On the other hand, Photofrin (97.03 ± 1.35% of remaining DPBF) and Radachlorin (83.11 ± 2.63% of remaining DPBF) showed lower singlet oxygen photogenerating ability than Photofrin under the same conditions. 

### 3.3. In Vitro Cytotoxicity Test

**Figure 3:**
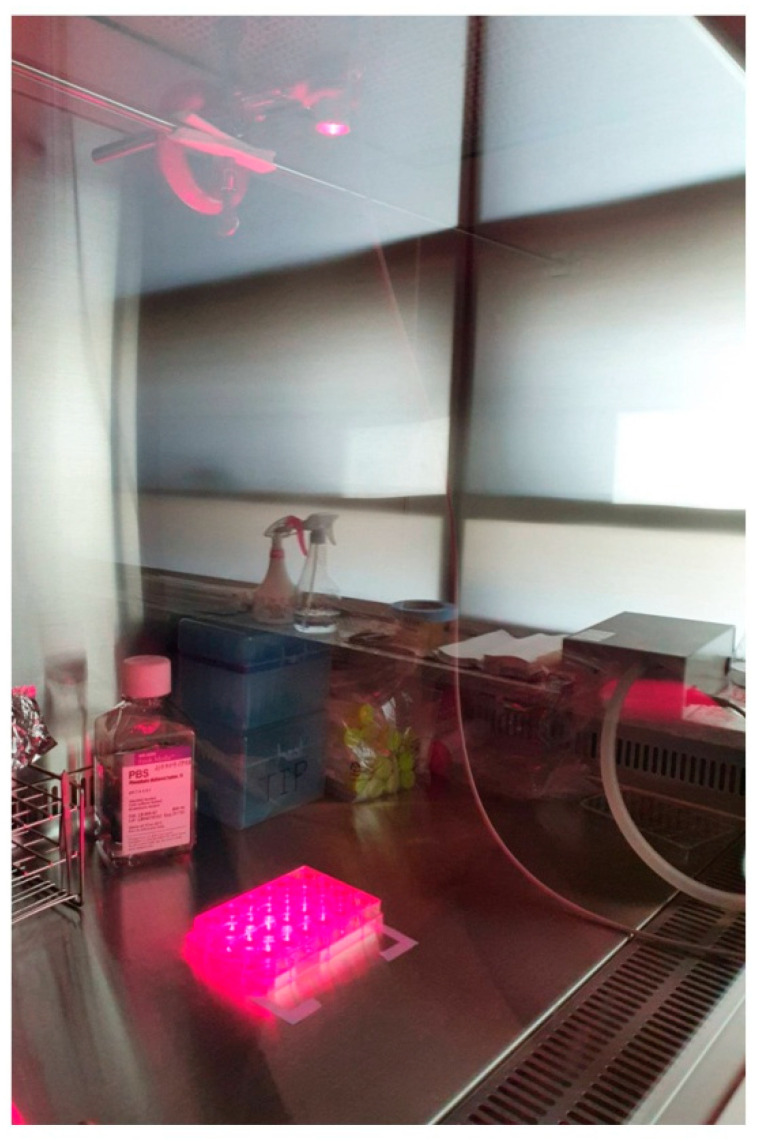
Scene of the in vitro PDT experiment. After 4 or 24 h of incubation with various concentrations (0, 0.1, 0.2 0.25, 0.4 and 0.5 μM) of photosensitizers under dark conditions, the cells were irradiated (5 mW/cm^2^, 400 s) with the proper wavelength (630 nm for Photofrin and 662 nm for Radachlorin and Photomed) of PDT lasers. Every in vitro irradiation was performed in the clean bench under dark conditions.

The cytotoxicity assay against SCC VII cells after various concentrations (0, 0.1, 0.2 0.25, 0.4 and 0.5 μM) of Photofrin, Radachlorin and Photomed treatment followed by no irradiation or light irradiation (5 mW/cm^2^, 400 s) was performed. The test was designed to evaluate whether Photomed itself is safe without PDT and whether Photmed is effective against cancer cells with PDT. The incubation time with the photosensitizers was set as a short time at 4 h and a long time at 24 h. Figure 3 shows the scene of the in vitro PDT experiment. Every light irradiation was performed in the clean bench to protect the cells from any contamination and under dark conditions to avoid the influence of any other light source.

**Figure 4:**
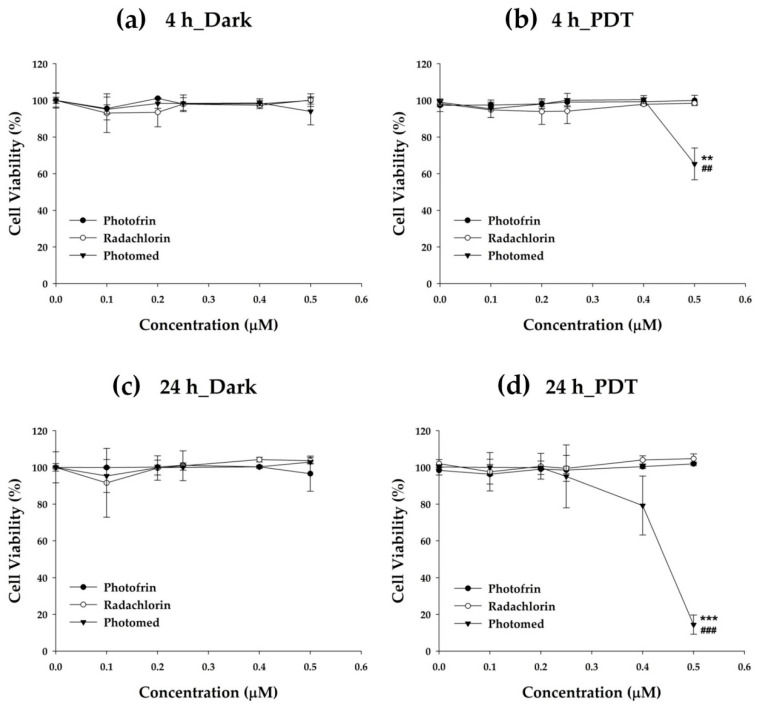
In vitro viability of SCC VII cells after various concentrations (0, 0.1, 0.2 0.25, 0.4 and 0.5 μM) of Photofrin, Radachlorin and Photomed treatment. (**a**) 4 h incubation of photosensitizers followed by no irradiation. (**b**) 4 h incubation of photosensitizers followed by light irradiation (PDT). (**c**) 24 h incubation of photosensitizers followed by no irradiation. (**d**) 24 h incubation of photosensitizers followed by light irradiation (PDT). Data are presented as means ± SD (*n* = 3). ** *p* < 0.01, *** *p* < 0.001, Photofrin vs. Photomed. ## *p* < 0.01, ### *p* < 0.001, Radachlorin vs. Photomed.

Figure 4 presents the cell viability data of Photofrin, Radachlorin and Photomed from four different conditions. Under dark conditions, every photosensitizer revealed no cytotoxicity at any concentration even during the long time (24 h) incubation. The photosensitizers including Photomed were proved to be safe as long as no light irradiation was treated. In addition, the light source condition was also checked for its safety. As shown in PDT groups, when the concentration of drug was 0 μM, no cytotoxicity was found. This indicates that the laser irradiation itself is not able to damage any cells without combining with photosensitizer administration. The antitumor efficacy of PDT against cancer cells may be determined by Figure 3. From the 4 h incubation data, at a 0.5 μM concentration, Photomed showed 65.33 ± 8.69% of cell viability while Photofrin and Radachlorin showed 99.98 ± 2.84% and 98.45 ± 0.62%, respectively. Photomed significantly inhibited the cancer cell survival with PDT compared to Photofrin (*p* = 0.0028) and Radachlorin (*p* = 0.0028). From the 24 h incubation data, Photomed showed more enhanced antitumor effect with PDT than the 4 h incubation. At a 0.4 μM concentration, though there was no significant difference between Photomed and either Photofrin or Radachlorin, Photomed showed decreased cell viability (79.20 ± 16.06%) while Photofrin and Radachlorin showed 100.42 ± 1.00% and 104.07 ± 2.21%, respectively. At a 0.5 μM concentration, Photomed showed strong cytotoxicity (14.43 ± 5.24% of cell viability) while Photofrin and Radachlorin showed no cytotoxicity at all (101.95 ± 0.91% and 104.73 ± 2.57% of cell viability, respectively). There were significant differences between Photomed and both Photofrin (*p* = 0.000009) and Radachlorin (*p* = 0.00001) when incubated for 24 h at a 0.5 μM concentration and treated with PDT.

**Figure 5:**
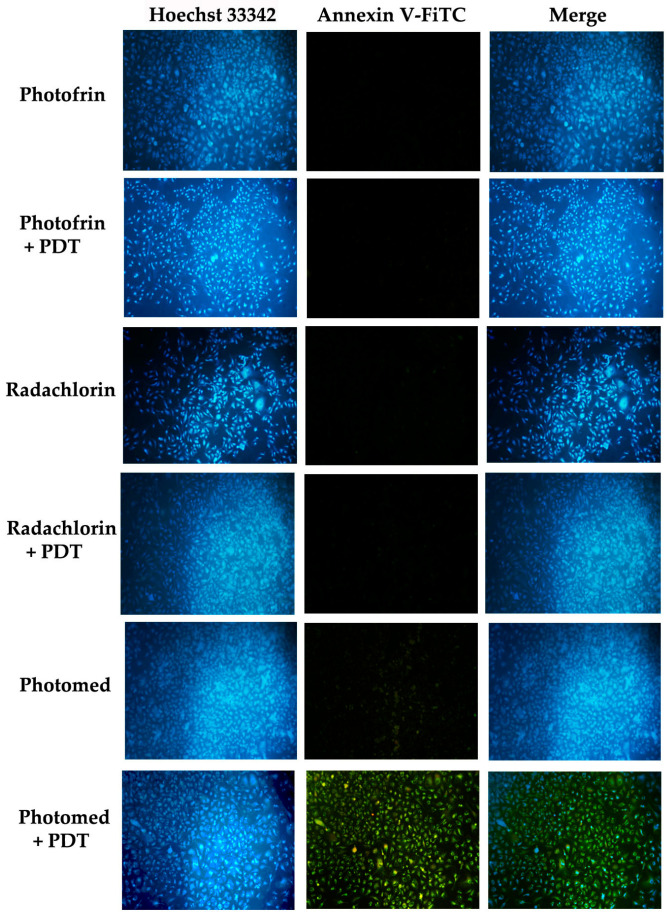
Fluorescence microscope analysis of apoptotic cells induced by PDT. Amounts of 0.5 μM of Photofrin, Radachlorin and Photomed were treated for 24 h followed by no irradiation or light irradiation (5 mW/cm^2^, 400 s). Hoechst 33342 staining (blue, first column) indicating nuclei and Annexin V-FITC staining (green, second column) indicating apoptotic cells by PDT. Magnification 100×.

In Figure 5, apoptotic cells induced by PDT were confirmed by fluorescence microscope analysis. Amounts of 0.5 μM of Photofrin, Radachlorin and Photomed were pre-treated in the cells for 24 h followed by no illumination or illumination with PDT lasers (630 nm for Photofrin and 662 nm for Radachlorin and Photomed) for 5 mW/cm^2^, 400 s. Hoechst 33342 staining indicated the nuclei part with blue fluorescence and Annexin V-FITC staining for apoptotic cells with a green signal. Concurrent with cell viability data after PDT, only Photomed-induced PDT revealed apoptotic cells.

Based on the results of in vitro cytotoxicity studies, Photomed was proven to be (1) a safe photosensitizer without laser irradiation and (2) the most effective photosensitizer with PDT against cancer cells compared to Photofrin and Radachlorin. The singlet oxygen photogeneration assay data (Figure 2) strongly support this.

### 3.4. In Vivo Anticancer Efficacy Study

In vivo anticancer efficacy study was performed using SCC VII tumor-bearing mice models. The tumor-induced mice were divided into the small-tumor group (tumor size ranging from 100 to 200 mm^3^) and the large-tumor group (tumor size ranging from 200 to 300 mm^3^) in order to evaluate whether Photomed-induced PDT is effective for not only the small-tumor group but also for the large-tumor group. This is a critical point since, for now, only patients who have small-sized tumors are able to receive PDT for their effective treatment. For lung cancer treatment with PDT, less than 1 cm of tumor size is recommended [25]. For the early stage of esophageal cancers, patients with tumor sizes less than 1 cm obtained a result of 100% complete regression after PDT; however, the patients with tumors of a size of 3.1–5.0 cm showed only 33% complete regression after PDT [26]. Various clinical reports state the importance of tumor size, which may be an important key to change the response rate after PDT treatment irrespective of the cancer type [27,28,29,30].

**Figure 6:**
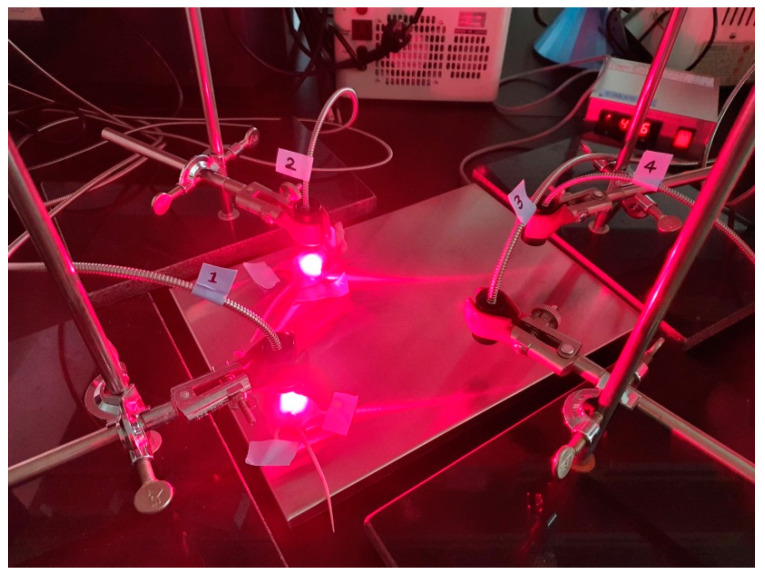
Scene of the in vivo PDT experiment. Tumor-bearing mouse models were established by subcutaneously injecting SCC VII cells to BALB/C nude mice. Three hours after intravenous injection (on day 0 and 7) of 1 mg/kg of photosensitizers, tumors were irradiated (400 mW/cm^2^, 500 s) with the proper wavelength (630 nm for Photofrin and 662 nm for Radachlorin and Photomed) of PDT lasers (on day 0 and 7). Every in vivo irradiation was performed on the heating pad set at 37 °C and under anesthesia.

Both the small-tumor group and the large-tumor group were then randomly divided into four groups: PBS, Photofrin, Radachlorin and Photomed, respectively. In this study, our design performed PDT twice to enhance the therapeutic effects against cancers. The first PDT was performed on day 0. PBS or phtosensitizers (1 mg/kg) were injected via the tail vein, and tumors were irradiated (400 mW/cm^2^, 500 s) 3 h after drug administration. One week after the first PDT, the second PDT was performed (on day 7) in the same way as in the first PDT. Figure 6 shows the scene of the in vivo PDT experiment. Every laser irradiation was performed with the animals lying down on a heating pad set at 37 °C in order to maintain the body temperature of the animal models during the treatment. After PDT treatment, the mice were housed in the dark for 24 h to avoid the influence of any other light source.

**Figure 7:**
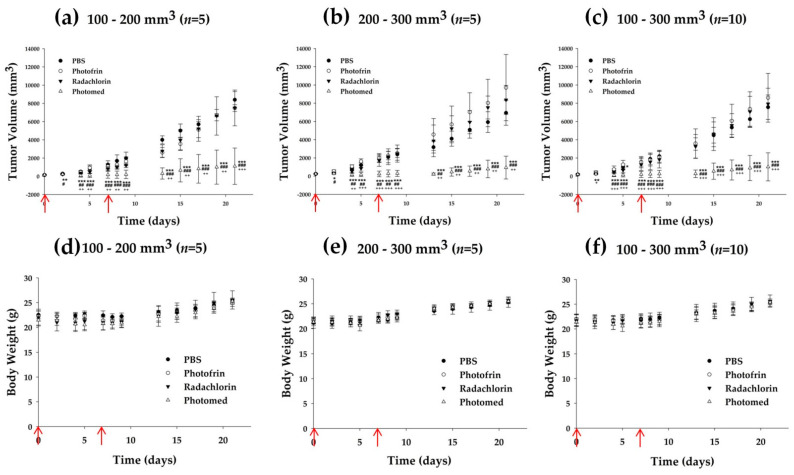
Tumor volume and body weight monitoring of SCC VII tumor-bearing mice after double (on days 0 and 7, red arrows) administration of PBS, Photofrin, Radachlorin and Photomed followed by light irradiation (400 mW/cm^2^, 500 s) (on days 0 and 7, red arrows) 3 h after each injection. (**a**,**d**) Small-tumor group (tumor size ranging from 100 to 200 mm^3^) (*n* = 5). (**b**,**e**) Large-tumor group (tumor size ranging from 200 to 300 mm^3^) (*n* = 5). (**c**,**f**) Small- and large-tumor combined group (tumor size ranging from 100 to 300 mm^3^) (*n* = 10). The tumor volume (mm^3^) was calculated using the following formula; (length × width^2^)/2. Data are presented as means ± SD. * *p* < 0.05, ** *p* < 0.01, *** *p* < 0.001, PBS vs. Photomed. # *p* < 0.05, ## *p* < 0.01, ### *p* < 0.001, Radachlorin vs. Photomed. + *p* < 0.05, ++ *p* < 0.01, +++ *p* < 0.001, Photofrin vs. Photomed.

The tumor volume and body weight of every mouse was monitored for 3 weeks after the first PDT. Figure 7 shows the tumor volume and body weight changes from each group. From the small-tumor group data (Figure 7a), every photosensitizer showed antitumor efficacy compared to PBS treated group. After the second PDT, every photosensitizer group represented stronger effects leading to a greater difference with the PBS group. Among the photosensitizer groups, the Photomed group showed superior PDT-mediated antitumor efficacy. Just after the first PDT, Photomed-mediated PDT significantly inhibited the tumor progression compared to PBS-, Photofrin- and Radachlorin-mediated PDT. On day 21, the tumor volume of the Photomed group was 1107.10 ± 1991.17 mm^3^, while those of the PBS, Photofrin and Radachlorin groups were 8398.99 ± 895.87 mm^3^ (*p* = 0.0000008, 7.59-fold compared to Photomed), 7484.61 ± 367.77 mm^3^ (*p* = 0.0007, 6.76-fold compared to Photomed) and 7500.51 ± 1959.97 mm^3^ (*p* = 0.000004, 6.77-fold compared to Photomed), respectively. In addition, interestingly, four out of five mice in the Photomed group in the small-tumor group showed a complete response from day 4. Among them, one mouse showed regeneration of the tumor on day 15, and one mouse showed it on day 19. The other two mice had no recurrence for 3 weeks. There was no complete response from any other group.

From the large-tumor group data (Figure 7b), in spite of double PDT treatments, the Photofrin and Radachlorin groups showed no antitumor efficacy. However, unlike other photosensitizers, the Photomed group revealed strong antitumor efficacy even in the large-tumor group. Same as the result in the small-tumor group, Photomed-mediated PDT showed a significantly improved antitumor effect compared to PBS-, Photofrin- and Radachlorin-mediated PDT right after the first PDT treatment. On day 21, the tumor volume of the Photomed group was 937.83 ± 1247.33 mm^3^, while those of the PBS, Photofrin and Radachlorin groups were 6937.64 ± 1357.36 mm^3^ (*p* = 0.0006, 7.40-fold compared to Photomed), 9710.51 ± 3631.50 mm^3^ (*p* = 0.004, 10.35-fold compared to Photomed) and 8359.33 ± 1577.62 mm^3^ (*p* = 0.0003, 8.91-fold compared to Photomed), respectively.

From small- and large-tumor combined group (Figure 7c), which is a mixture of the small-tumor group and the large-tumor group, on day 21, the tumor volume of Photomed group was 1022.47 ± 1540.83 mm^3^, while those of the PBS, Photofrin and Radachlorin groups were 7563.93 ± 1341.23 mm^3^ (7.40-fold compared to Photomed), 8597.56 ± 2669.36 mm^3^ (8.41-fold compared to Photomed) and 7929.92 ± 1709.90 mm^3^ (7.76-fold compared to Photomed), respectively. The Photomed group showed a significant difference from all the other PBS (*p* = 0.0000009), Photofrin (*p* = 0.000007) and Radachlorin (*p* = 0.0000007) groups. Taken all together, Photomed was proven to be (1) the most effective photosensitizer with PDT against tumor-bearing mice models compared to Photofrin and Radachlorin and (2) effective with PDT in treating not only small-sized tumors but also large-sized tumors. It should be highlighted that Photomed-mediated PDT may be applied even for the treatment of large tumors.

There was no severe change in body weight from every group (Figure 7d–f).

**Figure 8:**
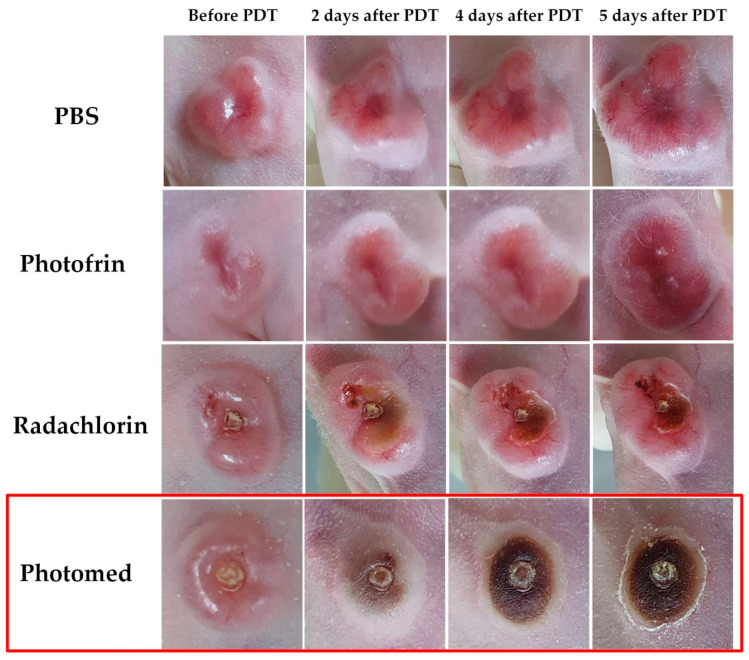
The representative tumor surface changes from SCC VII tumor-bearing mice before and after the first PDT with PBS, Photofrin, Radachlorin and Photomed.

Figure 8 represents the image of the representative tumor surface alterations from SCC VII tumor-bearing mice before and after the first PDT with PBS, Photofrin, Radachlorin and Photomed. The Photomed group showed a fast and complete response to tumors while the Photofrin and Radachlorin groups revealed slow and incomplete development of tumor necrosis. It may strongly support the fact that Photomed possesses the most superior anticancer efficacy among Photofrin, Radachlorin and Photomed when combined with PDT.

**Figure 9:**
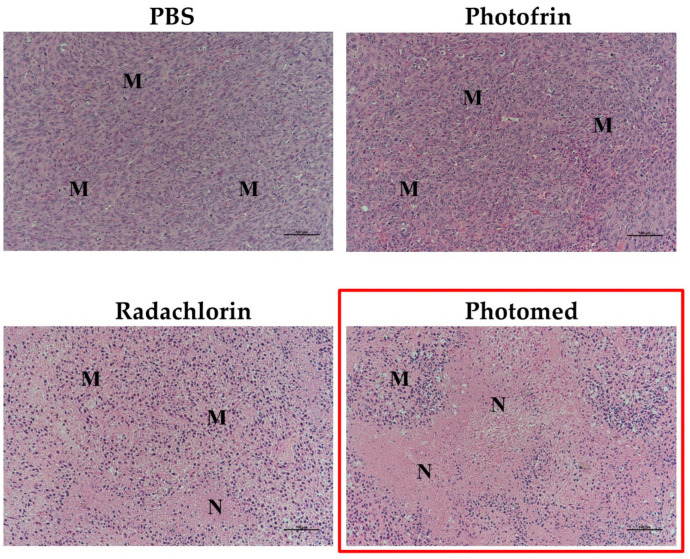
The representative histological analysis of tumor tissues from SCC VII tumor-bearing mice on day 10, after double (on days 0 and 7) intravenous injection of PBS, Photofrin, Radachlorin and Photomed with light irradiation (on days 0 and 7) 3 h after each administration (M: mitotic area, N: necrotic area). The scale bar in images represents 100 μm.

For the final in vivo evaluation, H&E stain of tumor tissues was performed on day 10 to observe the necrosis or apoptosis of tumors. As shown in Figure 9, Photomed-induced, PDT-treated tumor tissue displayed the most complete tumor cell death. The increased necrotic portion and the decreased mitotic area were found from the Photomed group. From this, another confirmation of the strong antitumor efficacy of PDT with Photomed was established.

From in vivo anticancer efficacy studies including tumor size monitoring, tumor surface monitoring and histological analysis, Photomed was proven to be the most effective photosensitizer with PDT to treat cancer when compared to Photofrin and Radachlorin.

Comparison of Photofrin, Radachlorin and Photomed is summarized in Table 1.

## 4. Conclusions

In the present study, the anticancer effect of PDT with a novel photosensitizer, Photomed, was compared with Photofrin and Radachlorin. Based on the in vitro and in vivo studies, Photomed was proven to be (1) a safe photosensitizer without laser irradiation, (2) the most effective photosensitizer with PDT against cancers compared to Photofrin and Radachlorin and (3) effective with PDT in treating not only small-sized tumors but also large-sized tumors. In conclusion, Photomed may contribute as a novel potential photosensitizer in PDT to treat cancers. Further studies should be performed since this study is an initial stage of preclinical studies.

## Figures and Tables

**Table 1:** Characteristics and preclinical data of Photofrin, Radachlorin and Photomed.

Photosensitizer	Class	Molecular Formula	Excitation Wavelength (nm)	Molar Extinction Coefficient (M^−1^ cm^−1^)	Relative Tumor Volume (at Day 21)
Photofrin	Porphyrin	C_34_H_38_N_4_NaO_5_	630	3000	6.76
Radachlorin	Chlorin	C_34_H_36_N_4_O_6_ C_33_H_34_N_4_O_5_ C_33_H_34_N_4_O_6_	662	34200	6.77
Photomed	Chlorin	C_38_H_42_N_6_O_3_	656	42463	1

## Data Availability

The data presented in this study are available on request from the corresponding author.
